# The Osteoporosis/Microbiota Linkage: The Role of miRNA

**DOI:** 10.3390/ijms21238887

**Published:** 2020-11-24

**Authors:** Massimo De Martinis, Lia Ginaldi, Alessandro Allegra, Maria Maddalena Sirufo, Giovanni Pioggia, Alessandro Tonacci, Sebastiano Gangemi

**Affiliations:** 1 Department of Life, Health and Environmental Sciences, University of L’Aquila, 67100 L’Aquila, Italy; lia.ginaldi@cc.univaq.it (L.G.); maddalena.sirufo@gmail.com (M.M.S.); 2 Allergy and Clinical Immunology Unit, Center for the Diagnosis and Treatment of Osteoporosis, AUSL 04 Teramo, 64100 Teramo, Italy; 3 Division of Hematology, Department of Human Pathology in Adulthood and Childhood “Gaetano Barresi”, University of Messina, 98125 Messina, Italy; aallegra@unime.it; 4 Institute for Biomedical Research and Innovation (IRIB), National Research Council of Italy (CNR), 98164 Messina, Italy; giovanni.pioggia@cnr.it; 5 Clinical Physiology Institute, National Research Council of Italy (IFC-CNR), 56124 Pisa, Italy; atonacci@ifc.cnr.it; 6 School and Operative Unit of Allergy and Clinical Immunology, Department of Clinical and Experimental Medicine, University of Messina, 98125 Messina, Italy; gangemis@unime.it

**Keywords:** osteoporosis, microbiota, microRNA, bone metabolism, gene expression, osteoblast, diet

## Abstract

Hundreds of trillions of bacteria are present in the human body in a mutually beneficial symbiotic relationship with the host. A stable dynamic equilibrium exists in healthy individuals between the microbiota, host organism, and environment. Imbalances of the intestinal microbiota contribute to the determinism of various diseases. Recent research suggests that the microbiota is also involved in the regulation of the bone metabolism, and its alteration may induce osteoporosis. Due to modern molecular biotechnology, various mechanisms regulating the relationship between bone and microbiota are emerging. Understanding the role of microbiota imbalances in the development of osteoporosis is essential for the development of potential osteoporosis prevention and treatment strategies through microbiota targeting. A relevant complementary mechanism could be also constituted by the permanent relationships occurring between microbiota and microRNAs (miRNAs). miRNAs are a set of small non-coding RNAs able to regulate gene expression. In this review, we recapitulate the physiological and pathological meanings of the microbiota on osteoporosis onset by governing miRNA production. An improved comprehension of the relations between microbiota and miRNAs could furnish novel markers for the identification and monitoring of osteoporosis, and this appears to be an encouraging method for antagomir-guided tactics as therapeutic agents.

## 1. Introduction

### 1.1. General Consideration on Osteoporosis

Osteoporosis is a systemic disease of the skeleton characterized by decreased bone mineral density (BMD) and structural deterioration, resulting in an increased risk of fragility fractures. Menopause and aging are the most common causes of osteoporosis. Genetic predisposition as well as lifestyle and nutrition are factors related to its pathogenesis. Estrogen, parathyroid hormone, inflammatory cytokines, and vitamin D are regulatory factors in the bone remodeling process. The pathophysiological process underlying osteoporosis is the imbalance of bone remodeling with increased bone resorption and consequent bone loss [1] as bone is a plastic tissue undergoing continuous remodeling.

Osteoblasts, derived from mesenchymal stromal cells of the bone marrow, are the precursors of osteocytes and perform bone-forming functions, producing matrix proteins and mineralization, whereas osteoclasts stem from mononuclear-phagocytic cells and function as bone resorbing cells. The balance between formation and resorption is essential for bone health [2].

Several diseases and the use of various types of drugs, in particular steroid therapies, can induce bone resorption and osteoporosis [3,4]. Estrogen deficiency and inflammatory conditions are known to result in bone resorption, mainly through the increased production of inflammatory cytokines, such as interleukin (IL)-1, IL-17, tumor necrosis factor (TNF)α, and receptor activator for nuclear factor-κB (RANKL) in the bone marrow, which induce an increase in osteoclast production, activation, and survival [5,6,7,8]. T helper (Th)17 lymphocytes play a central role in the process of accelerated bone loss in menopause [9]. Regulatory T cells (Tregs), which exert suppressive functions on the production of effector cytokines, intervene in the control of bone resorption through the production of osteoprotective cytokines, including Transforming Growth Factor (TGF) β1, IL-4, and IL-10, and the downregulation of osteoclast formation [10,11,12].

### 1.2. The Microbiota 

The microbiota is composed of trillions of microbial organisms, including bacteria, fungi, and viruses, living symbiotically with the host by increasing the absorption of nutrients from ingested food, as well as counteracting the colonization of pathogenic bacteria. With the advent of rapid sequencing technologies, many different bacterial species have been identified in the body, with approximately 100 times more bacterial genes (microbiome) than host genes [2]. The microbiota varies from person to person; however, there are four main classes of bacteria primarily represented in most normal subjects: *Firmicutes*, *Bacteroides*, *Proteobacteria*, and *Actinobacteria*, with *Bacteroidetes* and *Firmicutes* comprising over 90% of the phylogenic categories [13]. The microbiota can even be considered our largest organ, and recently, in addition to intestinal function control, other physio-pathological roles of the microbiota have emerged, including immune regulation, cancer development control, and bone remodeling [14,15].

### 1.3. Microbiota and Osteoporosis 

**Figure 1:**
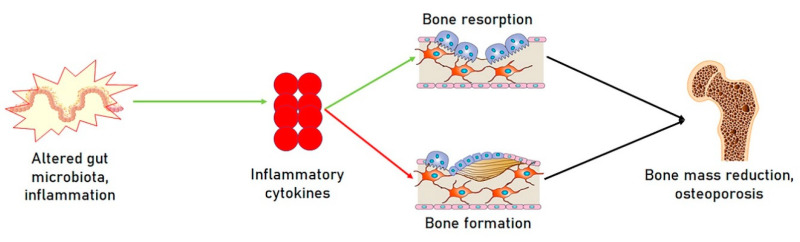
Relationship between gut microbiota and bone mass. An alteration of the gut microbiota can lead to the over-production of inflammatory cytokines, in turn promoting the activation of osteoclasts and bone, leading to bone resorption and inhibiting bone formation, ultimately driving to bone mass reduction and osteoporosis.

Microbiota and the host interact with each other in a dynamic equilibrium that influences the bone mass. Research established that both the immune system and the microbiota play fundamental roles in bone homeostasis, and in addition to the term “Osteo-immunology”, which refers to the crosstalk between the immune system and bone remodeling [16], the new term of “Osteo-microbiology”, meaning the functional relationship between the microbiota and bone, has been coined [17]. The microbiota modulates immune functions [18], and specific strains of intestinal microbes act on the cells of the immune system by modulating intestinal as well as systemic immune responses, thus, affecting distant organs and systems [19,20], such as the bones. The microbiota is able to intervene on other pathogenetic moments of osteoporotic disease (Figure 1).

Here, we address the relationships between microbiota and osteoporosis and, subsequently, the role of microRNAs (miRNAs) in this specific network. Several studies [20,21,22,23] have shown how the microbiota is closely related to the bone metabolism and the absorption of nutrients and minerals essential to the health of the skeleton. It is, therefore, not surprising that the development of osteoporosis is influenced by the microbiota.

The relationship between the microbiota and bones was first described few years ago by Sjogren et al. [24], who demonstrated that mice raised in germ-free conditions showed increased trabecular bone mass compared to controls and that the colonization with gut flora from conventionally raised mice was able to reverse this bone phenotype. They also found lower CD4+ T cell numbers and decreased TNF-a levels in the bone marrow from germ-free mice, associated with decreased osteoclast precursors and higher bone mass, suggesting that commensal gut microbiota decreases the bone mass by stimulating bone resorption and inhibiting bone formation [24]. 

Further studies showed that, in mice, the short-term administration of antibiotics that specifically reduce intestinal bacteria resulted in increased bone mass, and ovariectomy-induced bone loss in mice could be partially prevented by the administration of tetracyclines [25,26]. The antibiotic treatment, therefore, influenced the bone mass through modifications of the microbiota. In addition to reducing the microbiota, antibiotics also alter the composition, decreasing the diversity of the microbial taxa present in the intestine. Both the quantity and diversity of the intestinal bacterial load are likely to contribute to the mechanisms of regulation of bone mass by the microbiota. The colonization of germ-free mice with stool samples from malnourished children exhibiting an immature microbiota resulted in increased cortical bone density, shorter bones, and stunted body growth, suggesting that bone anabolic effects can be induced by an immature microbiota [27].

Different mechanisms of dysbiosis inducing osteoporosis have been hypothesized, such as a dysregulation of the immune–inflammatory axis [28]. Gut-mediated inflammation, with the intervention of inflammatory cytokines, plays a role in the activation of osteoclasts, favoring the appearance of osteoporosis [29]. The gut microbiome mediates osteoporosis pathogenesis by largely involving the immune system. *Clostridium* promotes the accumulation of Tregs, which are inhibitors of osteoclast differentiation, in the lamina propria of the colon [30]. A lack of *Clostridium* strains caused a reduction in Foxp3 Treg levels with an increase in bone loss [31]. 

T lymphocytes and osteoclast formation can be challenged by *Lactobacillus reuteri*. Osteoclastogenic Th17 cells can be differentiated due to an intestinal flora imbalance. Finally, Th17 differentiation can be promoted by mouse commensal segmented filamentous and human commensal bacteria [32]. B lymphocytes also regulate the function of bone cells by controlling the RANKL/osteoprotegrin (OPG) proportion via the phosphoinositide 3-kinase/protein kinase B (Akt)/mammalian target of rapamycin (mTOR) signal transduction pathway. 

Intestinal flora controls the mTOR transcription factors, thus, affecting B-cell development and, as a consequence, OPG production [33]. The decoy receptor OPG, in addition to the direct RANKL inhibition, inhibits osteoclastogenesis by modulating autophagy-related genes and AMP-activated protein kinase/mTOR/p70S6K signaling [34]. The microbiota modulates the production of insulin-like growth factor 1 (IGF-1), which is a regulator of bone remodeling [35], and microbiota dysregulations have been found to correlate with increased inflammatory responses and bone resorption [36,37]. 

However, an alteration of the microbiota could have other effects capable of mediating the onset of osteoporosis. The microbiota regulates the transport and absorption of nutrients necessary for the growth and maintenance of skeletal health and many metabolic functions as well as the production of various hormones, such as sex steroids, which play critical roles in skeletal turnover, and are influenced by the intestinal flora. The microbiota also affects bone health by regulating the metabolism of serotonin and vitamin D. 

In particular, vitamin D plays central roles in the bone metabolism, regulating the calcium channel function and promoting the intestinal absorption of both calcium and phosphorus as well as bone calcification [38]. In old age, there is an altered intestinal response to vitamin D and a reduced absorption of calcium associated with intestinal dysbiosis. In turn, intestinal dysbiosis can affect the absorption of calcium and vitamin D contributing to the development of osteoporosis. 

Vitamin D deficiency appears to induce a decrease in the ratio of *Firmicutes* to *Deferribacteres* in the gut and intestinal inflammation. Interestingly, the proportion of *Firmicutes* and *Deferribacteres* can be rebalanced by the administration of vitamin D, and colon inflammation also improved after vitamin D and/or antibiotic treatments [39]. In addition to vitamin D, vitamin B12 and folates, which are involved in bone turnover, are also regulated by the intestinal flora. Folic acid is involved in the metabolism of homocysteine, an amino acid produced during the metabolism of methionin. 

An altered gut microbiome can reduce folic acid absorption in the jejunum, leading to hyperhomocysteinemia, which, in turn, induces extracellular bone matrix degradation and decreases the bone mineral density [40]. Gut bacteria also affect the brain–gut axis by regulating the neurotransmitter serotonin (5-HT) [41]. Gut-derived 5-HT decreases bone formation, while brain-derived 5-HT has the opposite effect of increasing bone formation [42]. The expression of the rate-limiting enzyme 5-HT tryptophan hydroxylase-1 (TPH-1) in germ free mice was decreased [43].

The intestinal flora may also affect bone formation or destruction by modulating nitric oxide (NO) production. The biosynthesis of NO is known to be limited by nitric oxide synthase (NOS). Micro-organisms can promote the bond of pathogenic bacteria or bacterial lipopolysaccharide-inducible transcription factor nuclear factor (NF-kB) to the inducible nitric oxide synthase (iNOS) promoter, thus, upregulating iNOS transcription. iNOS stimulates osteoclast production by increasing the levels of RANKL. Vitamin D regulates endothelial NOS: it positively regulates NO, which, in turn, can influence the vitamin D actions on osteoblasts [44].

The alteration of the vitamin D receptor led to increased *Eggerthella* abundance and other unfavorable alterations in the intestinal microbiota in murine models [45]. Vitamin D levels were associated with a decrease in the relative abundance of *Escherichia/Shigella*. Microbes belonging to the phylum *Firmicutes*, including species from the genus *Veillonella*, which is decreased in osteoporotic patients, metabolize isoflavone diadzin to the estrogen analogue equol, suggesting that a reduction in *Veillonella* may lead to a lack of inhibition of bone resorption, through lower equol production [46]. These considerations support the concept that specific genera within the gut influence the bone metabolism in the host, subsequently affecting bone health [47].

### 1.4. miRNAs and Osteoporosis

MicroRNAs (miRNAs or miRs) are a set of small endogenous non-coding RNAs of 18–25 nucleotides that regulate gene expression through base complementarity between the seed region of the miRNA and the 3′-untranslated region (UTR) of the target mRNA. Corresponding to the quantity of complementary mRNA, miRNA connections can provoke mRNA translational degradation, repression, or both [48]. miRNAs can interfere in the onset of numerous pathologies, such as asthma, cancer, and inflammatory bowel disease [49,50,51].

They have an essential action in the natural bone growth, and, in a previous study, we reported that a specific miRNA profile existed in subjects with bisphosphonate-related osteonecrosis of the jaw with respect to control subjects. In these subjects, altered miRNAs were aimed at several genes and metabolic pathways involved in bone reabsorption, mineralization of the bone matrix, the calcium ion metabolism, and differentiation of bone tissue [52].

**Figure 2:**
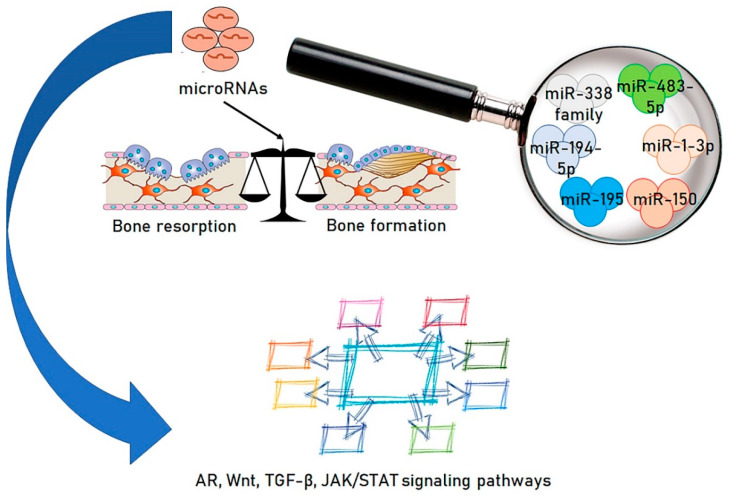
Selected microRNAs are known for balancing between bone resorption and formation, having a role in osteoporosis and for targeting several signaling pathways, including Androgen receptor (AR), Wnt, TGF-β, JAK/STAT signaling pathways.

Numerous experimental studies have demonstrated that miRNAs are also implicated in the onset of osteoporosis, principally in modulating the equilibrium between bone construction and bone reabsorption and osteoblast differentiation [53,54] [Figure 2]. Bioinformatics-based analyses have reported the existence of miRNA expression patterns correlated to postmenopausal osteoporosis [55,56].

For instance, Seeliger et al. executed microarray analysis from subjects with osteoporotic hip fractures and subjects with non-osteoporotic hip ruptures. They stated that five miRNAs were increased in the bone tissue and in the serum of subjects with osteoporotic fractures with respect to the subjects with non-osteoporotic fractures [57], and numerous other investigations confirmed that distinctive circulating miRNAs are correlated to osteoporosis [58,59,60,61,62,63,64].

In this regard, the analysis of Li et al. evaluating the miRNA levels of samples of postmenopausal women with osteoporosis appears particularly interesting. A total of 331 miRNAs were recognized as differently expressed miRNAs with respect to the control subjects. Among these, 122 miRNAs were increased, while 209 miRNAs were decreased. More than one hundred genes were identified as the objectives of these miRNAs. The Kyoto Encyclopedia of Genes and Genome analysis determined that the miRNAs primarily targeted pathways, such as the androgen receptor signaling pathway, wnt signaling pathway, TGF beta signaling pathway, and Janus kinase/signal transducers and activators of transcription (JAK-STAT) signaling pathway [65]. 

Certain specific miRNAs appear to be markedly relevant in the genesis of osteoporotic disease. A cross-sectional analysis enrolled 352 subjects, and a diagnosis of osteoporosis was made for 95 females and 30 males with BMD assays. The authors reported that miR-195 was considerably reduced in females, while miR-150 and miR-222 were substantially increased in males. In females, advanced age and decreased miR-195 were major risk elements for reduced BMD, while a decrease of miR-150 was a relevant risk element for osteoporosis [66]. 

miR-195 appertains to the miR-15 family, which is generated by stress and stimulated in numerous pathologies [67,68,69,70], and a correlation with bone metabolism has also been described [71,72]. miR-195 blocks the growth of chondrocytes by aiming at the G protein-coupled receptor kinase interacting protein-1 (GIT1), a central controller of bone mass in vivo by modulating osteoclast function [71]. Grunhagen et al. stated that miR-195-5p modifies the gene controlling system of osteoblast differentiation [72].

A different significant miRNA is miR-1-3p. Gu et al. stated that it was substantially reduced in the bones of osteoporotic subjects. Secreted frizzled-related protein 1 (SFRP1) was reported as a target gene of miR-1-3p. Their results demonstrated that the production of SFRP1 was inversely related with miR-1-3p in osteoporotic subjects. The increase of miR-1-3p augmented osteogenesis and reduced the adipogenesis of mesenchymal stem cells, while the in vivo reduction of miR-1-3p augmented the generation of SFRP1 and decreased bone formation [73]. 

miRNAs were stated to have relevant effects in controlling osteoclast differentiation. Research reported that an increase of miR-125a-5p augmented osteoclast differentiation through blocking TNFRSF1B expression [74], while Zhou et al. demonstrated that the increase of miR-100-5p avoided bone loss in ovariectomized animals through reducing the production of FGF-21 and osteoclast activeness [75].

Studies also showed that the miR-338 family was increased in postmenopausal osteoporotic women, and an estrogen-supported positive feedback (Runx2/Sox4/miR-338) loop was able to control osteoblast differentiation [76]. Finally, augmented concentrations of serum miR-483-5p and miR 194-5p have been demonstrated in different populations of osteoporotic subjects. [77,78]. 

Circulating miRNAs may be also a possible instrument for examining the effect of drugs on the osteoporosis. Patients with postmenopausal osteoporosis demonstrated a reduction in the serum amount of miR-33-3p after 3 months and miR-133a after 12 months of teriparatide administration [79]. Osteoporotic women demonstrated an increase in the serum concentrations of miR-497-5p and miR-181c-5p after treatment [80].

Finally, miRNAs could also play a role in the treatment of osteoporosis as miR-214-5p was reported to have an essential action in the adipogenic differentiation of bone marrow mesenchymal stem cells, and it might be a possible drug for osteoporosis [81]. 

## 2. Microbiota and miRNAs; A Novel Functional Axis

Current searches on the microbiota indicate its participation in the onset of different diseases through modulating the microbiota–gut axis, microbiota–brain axis, microbiota–liver axis, microbiota–lung axis, and microbiota–vascular axis [82]. Numerous experiments have also demonstrated the presence and actions of a microbiota–bone axis capable of inducing the onset of osteoporotic disease. The systems by which an altered microbiota can participate in the progression of osteoporosis diseases are manifold, and, among these, a fundamental moment could be constituted by the ability of the microbiome to intervene in the expression and functioning of miRNAs.

In osteoporosis, *Firmicutes* were significantly increased while *Bacteroidetes* were significantly decreased. The *Firmicutes/Bacteroidetes* ratio correlates negatively with the BMD, whereas an abundance of *actinobacteria* phylum members, such as *Bifidobacteriaceae*, positively correlates with the BMD [25]. In subjects with a normal BMD, *Bacteroides, Faecalibacterium,* and *Prevotella* represented more than half of the bacterial community, while, in patients with osteoporosis and osteopenia, 5 and 11 genera, respectively, constituted 50% of the bacterial community [83]. 

The genera *Parabacteroides*, *Blautia*, and *Ruminococcaceae* also differed significantly between osteoporotic patients and controls. Colonization by *Firmicutes* and the increase in the biodiversity in the intestinal bacterial flora were associated with increased local and systemic inflammatory responses, and responsible for the differentiation of osteoclasts from monocytic precursors in the bone marrow and their activation in the bone [22].

**Figure 3:**
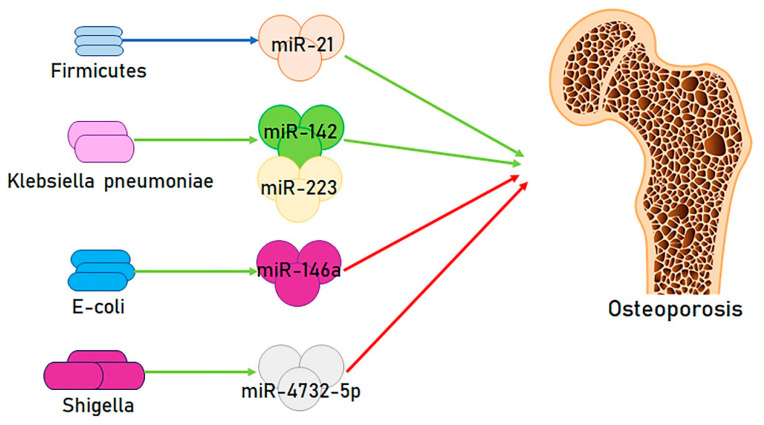
Simplified view of the action of the major intestinal bacteria on osteoporosis through microRNA regulation: (**i**) *Firmicutes* are known to modify miR-21 expression, associated with osteoporosis; (**ii**) *Klebsiella pneumoniae* drives to increased miR-142 and miR-223, in turn increased in osteoporosis; (**iii**) *E-coli* leads to an increase in miR-146a concentration, able to challenge osteoporosis by promoting osteogenesis; (**iv**) *Shigella* promotes increased miR-4732-5p concentrations, in turn associated with significant cell growth, also challenging osteoporosis.

Although data in the literature are sometimes conflicting, it has been shown that *firmicutes* were able to modify the expression of miRNAs associated with osteoporotic disease, such as miR-21 [84], and this miRNA has been recognized as having a role in the genesis of osteoporosis. In a paper, among 83 tested miRNAs, miR-21-5p concentrations were reported to be higher in the serum of osteoporotic subjects with respect to non-osteoporotic subjects (both with bone ruptures) [57], and this increase was confirmed by different reports in cohorts of fractured postmenopausal osteoporotic subjects [85,86] Figure 3.

Chen et al. evaluated the pattern of expression of 150 serum miRNAs in osteoporotic subjects and in a group of age-matched controls, and they found that six miRNAs were decreased, while five miRNAs comprising miR-21-5p were increased in the serum of osteoporotic subjects [87].

Regarding the systems via which miR-21 could exercise its effects on osteoporosis, researchers demonstrated that an increase of miR-21 augmented RANKL generation and reduced TGF-Beta 1 and OPG concentrations, and this was able to provoke an augment of RANKL/OPG ratio with a rise of bone reabsorption and reduction of BMD, producing osteoporosis [88].

*Klebsiella* and *Lachnoclostridium* were also found to be more plentiful in osteoporosis than in a normal subject group [22]. Research reported that, after the intratracheal administration of *Klebsiella pneumoniae*, several miRNAS, including miR-223/142, were markedly increased in the serum and bronchoalveolar lavage fluid, and a variation of the expression of this miRNA was reported in osteoporotic subjects [89]. miR-223-3p and other miRNAs were described as more augmented in osteoporotic subjects than in non-osteoporotic subjects (both with fractures), and the Receiver operating characteristic (ROC) analysis demonstrated the relevant capability of these miRNAs in discriminating osteoporotic from non-osteoporotic fractures [57]. A study also determined that miR-223 contributes to the calcification process by networking with osteoblasts and osteoclasts [90]. 

A different situation appears to be present with respect to *Clostridium*. Patients with clostridium infection had greater concentration levels of fecal miR-1246, while no modification was observed in serum samples. In any case, there are currently no studies that correlate alterations of this miRNA with the onset of osteoporosis [91].

Das et al. identified other taxa-specific differences in the gut microbiota profiles associated with normal bone mineral density, osteopenia, and osteoporosis that could present a link with miRNA expression. These genera could represent potential biomarkers and future therapeutic targets in high risk cohorts of osteoporotic patients. *Escherichia/Shigella* and *Veillonella* were more abundant in subjects with osteopenia compared with those with osteoporosis [92], and both *Escherichia* and *Shighella* are capable of modifying miRNAs involved in the genesis of osteoporosis. 

A study evaluated and confronted miRNA modifications of human epithelial and human monocytic THP-1 cells stimulated by the enteropathogenic *Escherichia coli* (EPEC) strain E2348/69 (O127:H6) and the probiotic strain *Escherichia coli Nissle* 1917 (EcN) (O6:K5:H1). THP-1 cells demonstrated a significant augment in miR-146a production, with a greater augment after EcN infection and a minor augment after EPEC infection [93]. 

An increased production of miR-146a was able to block the osteogenic capability of bone marrow stromal stem cells (BMSCs), while inhibiting miR-146a partly reverted the osteogenesis insufficiency under TNF-α treatment. Regarding the mechanism of action, miR-146a reduced Smad4 production by connecting to a part positioned in the Smad4 3′-untranslated region, and reestablishment of Smad4 inverted the repressive actions of miR-146a on osteogenesis [94]. These findings suggest that an inflammatory milieu is able to block osteogenesis through an increase of miR-146a and a decrease of Smad4. Research also demonstrated that polymorphisms of miR-146a were correlated with osteoporotic vertebral compression ruptures in postmenopausal women [95].

A therapeutic modification of miR-146a may be a possible approach to increase osteogenesis in the context of osteoporosis. This possibility was confirmed by the fact that a miR-146a knockout safeguarded bone loss in an animal experimental model of estrogen-deficient osteoporosis, and miR-146a blocked osteoblasts and osteoclast actions in vitro and in vivo. MiR-146a^−/−^ mice exhibited the same bone mass as the wild type (WT) but showed a greater bone turnover than the WT. However, miR-146a^−/−^ animals displayed an augment in BMD after experiencing ovariectomy with respect to animals exposed to sham operations. Osteoclast functions were also modified in the miR-146a^−/−^ animals subjected to estrogen insufficiency, which was contrary to the increased bone resorption capability of the WT [96]. Thus, miR-146a has a central action in estrogen insufficiency-caused osteoporosis, and the reduction of this miRNA offers skeleton defense.

However, not all the data in the literature appear to be univocal. In a study, the concentrations of miR-146a were estimated in the plasma of 120 postmenopausal subjects who were separated into three groups: normal, osteopenia, and osteoporosis. The modifications of the miR-146a concentrations in plasma among the three sets were not significant [97].

Instead, the production of endogenous miR-4732-5p and miR-6073 were augmented throughout *Shigella* infection [98]. Although there are no specific studies on the action of these miRNAs on the onset of osteoporosis, research demonstrated that MiR-4732-5p considerably increased the cell growth, colony formation, and migration of several types of cells [99].

*Actinomyces*, *Eggerthella*, *Clostridium XlVa*, and *Lactobacillus* were also more abundant in subjects with osteoporosis compared with the normal BMD group. These microorganisms modulate the host’s immune system and metabolism, and their functional analyses may provide insights into how the gut microbiota affects bone mineral density [92].

For example, *Actinomyces* are involved in the development of osteonecrosis of the jaw induced by bisphosphonates, and antimicrobial therapy targeting this organism has been proposed for its management [100]. Interestingly, *Clostridium XlVa* is a relevant producer of the short chain fatty acid butyrate, which stimulates bone formation, and is also a potent inducer Tregs, which, in turn, regulate bone homeostasis [101]. 

Both *Actinomyces* and *Lactobacillus* are able to modify miRNA expression. Naqvi et al. evaluated the initial (4 h) miRNA reaction of human monocytic THP1-derived macrophages stimulated with lipopolysaccharide (LPS) originating from the pathogen *Aggregatibacter actinomycetemcomitans* (Aa). Aa LPS determined the au-augmented production of miR-146a. This stimulation caused the release of a great quantity of TNF-α, that was associated with augmented concentrations of both pre- and mature miR-146a, which is capable of influencing the bone metabolism [102]. 

As far the actions of *Lactobacillus acidophilus* and *Bifidobacterium bifidum* on miRNAs production, a recent paper clarified their influence on the expression of miR-135b, 26b, 18a, and 155 and their target genes, comprising KRAS, APC, PU.1, and PTEN. The data demonstrated that the production of the miR-135b, miR-155, and KRAS was increased [103], and miR-135a-5p is otherwise produced between normal subjects and osteoporotic subjects with fractures. Research also demonstrated that employing the support vector machine algorithm classification 135a-5p could discriminate between the normal subjects and fractured patients, and the area under the curve was 0.9722 with 95% CI 0.8885–1.056 [104].

As seen above, miRNA-155 is a diverse miRNA stimulated by *Lactobacillus acidophilus* and *Bifidobacterium bifidum*. An osteoporosis animal experimental model was projected to evaluate the relationship between bone density and the amount of miR-155 in osteoclasts. Animals with osteoporosis showed reduced BMD and bone tension, and an increased production of miR-155. Down-regulation of miR-155 provoked a reduction of TNF-α, RANK, IL-1beta, M-CSF, TRAP, and Bcl-2, and an increase of the leptin receptor with an inhibition of the cell proliferation and bone resorption of osteoclasts [105]. Other data proposed that miR-155 reduction stimulated osteogenic differentiation of hBMSCs under high glucose and free fatty acid conditions by aiming at the silent information regulator 1. Blocking miR-155 may offer a novel therapeutic approach for the therapy of osteoporosis [106].

Finally, several data suggested that miR-26b also stimulated BMSC osteogenesis by triggering the canonical Wnt signal pathway, indicating that miR-26b might be employed as a possible therapeutic factor of osteoporosis [107].

An overall view of the miRNAs, functions, and target genes involved in the whole process is displayed in Table 1.

## 3. Modifying the Microbiota/miRNAs Axis: A New Approach to Osteoporosis Therapy

Nutritional supplementation with probiotics, i.e., selected live microorganisms capable of exerting positive effects for the health of the host, provided in adequate quantities and for sufficient periods, could find use in the therapy of osteoporosis [108]. Probiotics are essentially harmless and beneficial bacteria of the microbiota. The genes of these bacteria encode factors capable of governing the regulation of a wide spectrum of functions not only of the intestine itself but also of other organs. 

Through the regulation of vitamins, branched-chain fatty acids, and short chain fatty acid (SCFAs), they control the functioning of several systems. Numerous studies describe the positive effects of probiotics on bone mass in animals and humans [109,110]. In patients with osteoporosis receiving *Lactobacillus reuteri* orally, the loss of bone mineral density was significantly reduced compared to a placebo control group. A red clover extract (RCE), rich in isoflavone aglycones and probiotic lactic bacteria administered to patients with postmenopausal osteoporosis, improved bone turnover by promoting the production of estrogen metabolites that reduce bone loss [111]. The integration of probiotics can, therefore, be clinically useful to prevent bone resorption and osteoporosis. 

However, new studies have shown that it is possible to look at the problem from a different perspective. The link between osteoporosis, the microbiota, and miRNAs is supported by the literature, suggesting that miRNA can be modulated to modify the onset and progression of neoplastic disease via a modification of the microbiota obtained by the use of probiotics or dietary advice.

The diet influences the microbiota, in turn, regulating miRNA expression [112]. Then, diet and probiotics could modify the microbiota and such modifications can affect the synthesis of miRNAs, although a bidirectional relationship between microbiota and miRNAs cannot be ruled out.

For example, in the previous section, we reported the relevant role played by mir-21 in the genesis of osteoporosis. The nutritional change of miR-21 production was studied in several in vivo and in vitro analyses. A very potent epigenetic modulator of miR-21 may be the phenolic substance resveratrol, a compound generally present in red wine and peanuts, capable of reverting the dysbiosis in db/db mice typified by low amounts of *Bacteroides*, *Alistipes*, *Rikenella*, *Odoribacter*, *Parabacteroides*, and *Alloprevotella* [113].

Resveratrol is capable of modifying miR-21 expression in diverse cell culture models. Experiments employing the culture of U251 cells, cultured with resveratrol for 12 h, reduced miR-21 expression, and this was followed by the decrease in the generation of proinflammatory transcription factor NF-κB [114]. The reduction of miR-21 could have a beneficial effect on the progression of osteoporosis.

A different useful dietary substance could be curcumin, which is a polyphenol diferuloylmethane, extracted from curcuma (*Curcuma longa*) that has been demonstrated to have antioxidant and anti-inflammatory effects [115,116]. 

Recently, researchers assumed that curcumin could exercise regulative actions in the gastrointestinal tract, where elevated levels have been discovered after oral dispensation. It might be conjectured that curcumin acts on the gut microbiota, thus, explicating the paradox between its small bioavailability and its pharmacological effects [117]. Clinical reports stated that miR-21 and miR-155 production were reduced after the daily ingestion of curcumin [118,119].

In addition to the opportunity to change the levels of host-generated miRNAs, numerous food-derived exogenous miRNAs have been identified. This suggests that nutritional components themselves are a source of miRNAs that could regulate homeostasis and microbiota and intervene in several pathological conditions, such as osteoporosis [120].

Variation of gene expression by diet-originated miRNAs might be implicated in the interactions between microbiota, miRNAs, and osteoporosis, and this correlation could be bidirectional. Via an informatics methodology, Teodori et al. looked for suggestions that food-containing miRNAs—essentially implicated in the regulation of the inflammatory systems as the so called inflamma-miRNAs—may participate in the anti-inflammatory actions exercised by some foods via the variation of microbiota configuration in a bidirectional interaction. In particular, three different inflamma-miRNAs were evaluated: miR-155, miR-146a, and miR-21, miRNAS that are all implicated in the onset and in the progression of osteoporosis. 

The in silico analysis corroborated the possibility that these inflamma-miRNAs could regulate some metabolic pathways, such as the elongation of fatty acids, which are implicated in the regulation of microbiota structure, i.e., *oscillibacter*, *prevotella*, and *ruminococcus*, and vice versa. Dietary homologues to human miR-155, miR-21, and miR-146a were identified in eggs, cow milk, and cow fat, indicating that they may be capable of influencing, and possibly aggravating, inflammation correlated systems. If these results are confirmed, they will sustain the importance of a nutraceutical procedure for the treatment of osteoporosis [121].

Finally, a particularly fascinating field of investigation could be the study of changes in bone density during the growth period, and the intricate relationship present between the microbiota, diet, and miRNAs could even be accountable for BMD commencing in the early phases of development [122].

## 4. Conclusions

Several factors, including diet, antibiotics, and probiotics, impact the microbiota [123,124,125], which, in turn, affects the regulation of bone mass through a variety of different mechanisms. Some species of microbiota bacteria, by increasing the bioavailability of estrogen, exert positive effects on the skeleton by increasing bone mass with the help of prebiotics. The microbiota can increase the production of inflammatory cytokines from the immune system, which increases osteoclastogenesis. Metabolites produced by the microbiota, including short-chain fatty acids, influence the absorption of minerals essential for bone formation, and the microbiota modifies the intestinal permeability and enhances the promoting effect of vitamin D on the absorption of bone minerals [126]. 

A further mechanism through which the microbiome is able to affect the onset of osteoporosis could be its action on miRNAs. Several miRNAs are able to regulate the substances linked to the differentiation of osteoblasts in osteoporosis, promoting this event and, thus, challenging osteoporosis progression. On the other side, miRNAs could also inhibit the differentiation of osteoblasts and challenge the healing of osteoporosis [127,128].

Modulation, by increasing or reducing these miRNAs acting on microbiota, could help control the disease. Presently, the use of probiotics or diets that intend to control the microbiota, are thought to be a possible therapeutic approach to modify miRNA expression, influence BMD, and intervene in osteoporosis. This could be a low-cost and secure approach to re-establish a healthy status. 

Numerous reports demonstrated that several miRNAs could be modified by the microbiota and employed as prognostic or diagnostic markers for differentiating osteoporotic patients from non-osteoporotic subjects. In plasma samples from osteoporotic and osteopenia postmenopausal subjects, the miR-133a and miR-21 concentrations were, respectively, augmented and reduced with respect to healthy controls and both were correlated to the BMD [129].

In-depth knowledge of the mechanisms that regulate the relationships between the microbiome and miRNAs could open a new era in disease treatment and prevention. A huge series of novel research fields appears to be opening up in the context of the study on microbiota, osteoporosis, and non-coding genetic material other than miRNAs, such as long non-coding RNA (lncRNA) [130]. LncRNA expression in the gut forms a molecular signature that may unveil the classes of microbes, and Liang et al. proved the presence of a relation between lncRNA expression and gut microbes [131,132]. 

In conclusion, the pathogenic mechanisms of osteoporosis at the epigenetic level are becoming increasingly clearer and have led to epigenetic-related therapies for the treatment of osteoporosis [133].

Interventions on the microbiota to modify the expression of non-coding genetic material could represent a new frontier in the treatment of osteoporosis.

## Figures and Tables

**Table 1:** miRNAs, functions, and target genes involved in the osteoporosis/microbiota linkage.

miRNA	Target(s)	Function(s)	Reference(s)
miR-1-3p	SFRP1	Osteogenesis, adipogenesis, bone formation regulation	[73]
miR-21	RANKL, TGF-Beta 1, OPG	Bone reabsorption	[88]
miR-26b	Wnt pathway	Osteogenesis	[107]
miR-100-5p	FGF-21	Avoids bone loss	[75]
miR-125a-5p	TNFRSF1B	Increased osteoclast differentiation	[74]
miR-146a	Smad4	Osteogenesis blockade	[94,95]
miR-155	KRAS, TNF-α, RANK, IL-1beta, M-CSF, TRAP, and Bcl-2	Stimulation of cell proliferation	[105,106]
miR-195	GIT1	Blocks the growth of chondrocytes	[71,72]
